# Down-regulation of NF kappa B activation is an effective therapeutic modality in acquired platinum-resistant bladder cancer

**DOI:** 10.1186/s12885-015-1315-9

**Published:** 2015-04-29

**Authors:** Yujiro Ito, Eiji Kikuchi, Nobuyuki Tanaka, Takeo Kosaka, Eriko Suzuki, Ryuichi Mizuno, Toshiaki Shinojima, Akira Miyajima, Kazuo Umezawa, Mototsugu Oya

**Affiliations:** 1 Department of Urology, Keio University School of Medicine, 35 Shinanomachi, Shinjuku-ku, Tokyo 160-8582 Japan; 2 Department of Applied Biological Science, Tokyo University of Agriculture and Technology, 3-8-1 Harumi-cho, Fuchu-shi, Tokyo 183-8538 Japan; 3 Department of Molecular Target Medicine Screening, Aichi Medical University, 1-1 Yazakokarimata, Nagakute, Aichi 480-1195 Japan

**Keywords:** Bladder cancer, NF-κB, Paclitaxel, Platinum resistance, DHMEQ

## Abstract

**Background:**

No previous study has addressed the efficacy of NF-κB blockade when bladder tumors develop acquired resistance toward CDDP-treatments. We investigated the changes in NF-κB activation and therapeutic impact of NF-κB blockade by the novel NF-κB inhibitor dehydroxymethyl derivative of epoxyquinomicin (DHMEQ) in CDDP-resistant bladder cancer cells.

**Methods:**

Two human invasive bladder cancer cell lines, T24 and T24PR, were used. The T24PR cell line was newly established as an acquired platinum-resistant subline by culturing in CDDP-containing medium for 6 months. Expression of intranuclear p65 protein in the fractionated two cell lines was determined by Western blotting analysis. DNA-binding activity of NF-κB was detected by electrophoretic mobility shift assay. The cytotoxic effects and induction of apoptosis were analyzed in vivo and in vitro.

**Results:**

Intranuclear expression and DNA-binding activity of p65 were strongly enhanced in T24PR cells compared with those of T24 cells, and both were significantly suppressed by DHMEQ. Lowered cell viability and strong induction of apoptosis were observed by treatment with DHMEQ alone in these chemo-resistant cells compared with parent cells. As T24PR cells did not show dramatic cross-resistance to paclitaxel in the *in vitro* study, we next examined whether the combination of DHMEQ with paclitaxel could enhance the therapeutic effect of the paclitaxel treatment in T24PR tumors. Using mouse xenograft models, the mean volume of tumors treated with the combination of DHMEQ (2 mg/kg) and paclitaxel (10 mg/kg) was significantly smaller than those treated with paclitaxel alone (p < 0.05), and the reduction of tumor volume in mice treated with DHMEQ in combination with paclitaxel and paclitaxel alone as compared to vehicle control was 66.9% and 17.0%, respectively.

**Conclusion:**

There was a distinct change in the activation level of NF-κB between T24 and T24PR cells, suggesting strong nuclear localization of NF-κB could be a promising target after developing acquired platinum-resistance in bladder cancer.

## Background

Bladder cancer is one of the most aggressive epithelial tumors and is characterized by a high rate of early systemic dissemination. Patients with metastatic bladder cancer are routinely treated with cisplatin (CDDP)-based systemic chemotherapy, such as M-VAC (methotrexate, vinblastine, doxorubicin, CDDP) or GC (gemcitabine, CDDP) regimens. Such CDDP-based regimens have generally produced a complete or partial response in approximately 50-70% of patients [1,2]. However, tumors treated with CDDP finally acquire platinum resistance, and no standard of care exists when tumors develop after CDDP-treatments. These disappointing results have prompted an ongoing search for novel agents and multidrug combinations in this area.

NF-κB, a heterodimer consisting mainly of p65 and p50 proteins, functions as a transcription factor that induces inflammatory cytokines and antiapoptotic proteins. A growing body of evidence indicates that the activation of NF-κB is associated with resistance to apoptosis, expression of angiogenic proteins, and carcinogenesis due to its fundamental effects on cellular dedifferentiation and proliferation in malignancies [3,4]. DHMEQ, is a novel and potent NF-κB inhibitor [5] that binds to a Cys residue of p65 and acts at the level of nuclear translocation [6]. The mechanism by which DHMEQ inhibits activation of NF-κB is unique because DHMEQ inhibits NF-κB translocation from the cytoplasm to the nucleus [7]. Using this agent, we previously showed that inhibition of the NF-κB pathway led to a potent induction of apoptosis in renal cell cancer, bladder cancer, and prostate cancer cells [8-10], suggesting that the regulation of NF-κB may be a potent therapeutic target for urogenital cancer.

The aim of the present study was to investigate the efficacy of NF-κB blockade as a new modality for treating platinum-resistant advanced bladder cancers. Also, we evaluated the efficacy of other chemotherapeutic agents such as gemcitabine, paclitaxel and carboplatin as second line chemotherapy for CDDP-resistant bladder tumor cell lines. To the best of our knowledge, no study has ever addressed the impact of NF-κB blockade when bladder tumors develop acquired resistance toward CDDP-based treatments. Also, few studies have described the changes in NF-κB expression in such tumors. We believe that these results may highlight the importance of NF-κB regulation as well as the clinical potency of DHMEQ in the treatment of metastatic bladder cancer.

## Methods

### Cell lines and agents

Two human invasive bladder cancer cell lines, T24 and T24PR, were used. T24 cells were obtained from the American Type Culture Collection (Rockville, MD, USA). T24PR cells were established in our laboratory as an acquired platinum resistant cell line [11]. Briefly, T24 cells were grown and passaged upon reaching confluence in medium containing CDDP over a 6-month period to develop platinum resistance. All cells were routinely maintained in RPMI-1640 (Invitrogen, Carlsbad, CA, USA) supplemented with 10% fetal bovine serum (Dainippon Pharmaceutical, Tokyo, Japan), at 37°C in a humidified 5% CO_2_ atmosphere. DHMEQ, synthesized as described previously [10,12], was dissolved in dimethyl sulfoxide (DMSO) at a concentration of 10 mg/ml and stored at −20°C. This stock solution was diluted in culture medium to a final concentration of <0.1%. CDDP and paclitaxel were kindly supplied by Nippon Kayaku Co. (Tokyo, Japan). Gemcitabine and carboplatin were obtained from Wako Pure Chemical Industries (Osaka, Japan).

### Cell extracts and western blotting analysis

Proteins were extracted from the cytoplasm and nucleus separately using NE-PER nuclear and cytoplasmic extraction reagents (Pierce Biotechnology, Rockford, IL, USA) according to the manufacturer’s protocol. The extracted nucleus protein (20 μg) and cytoplasmic protein (20 μg) with sample buffer containing 2-mercaptoethanol was separated on 12.5% SDS-PAGE and transferred to a nitrocellulose membrane (Bio-Rad Laboratories, Hercules, CA, USA) and then incubated with 5% skim milk overnight. The membrane was then incubated overnight with primary antibodies against NF-κB p65 (Cell Signaling Technology, Beverly, MA, USA), Lamin A⁄C (Santa Cruz Biotechnology, Dallas, TX, USA), beta-actin (Sigma-Aldrich, St. Louis, MO, USA), Bcl-2 (Santa Cruz Biotechnology, Dallas, TX, USA) and survivin (Santa Cruz Biotechnology, Dallas, TX, USA). After incubation with appropriate secondary antibodies, signals were visualized using an ECL Western blotting system (Amersham, Piscataway, NJ, USA).

### Electrophoretic mobility shift analysis

A nuclear extraction kit (Affymetrix, Santa Clara, CA, USA) was used to prepare nuclear extracts and an EMSA “Gel Shift” kit (Affymetrix, Santa Clara, CA, USA) was used in gel shift assay. The binding reaction mixture contained 2 μL of nuclear extract (at a concentration of 2 μg/μL), 1 μL of poly (dI–dC), and biotin-labeled p65 probe in binding buffer (75 mM NaCl, 1.5 mM EDTA, 1.5 mM DTT, 7.5% glycerol, 1.5% NP-40, 15 mM Tris–HCl; pH 7.0). Samples were incubated for 30 min at 15°C in this mixture. DNA/protein complexes were separated from free DNA on a 6% non-denaturing polyacrylamide gel in 0.25 mM TBE buffer. The gel was transferred to a nylon membrane and detected using streptavidin-HRP and chemiluminescent substrate. The following sequence was used as a p65 probe (Affymetrix, Santa Clara, CA, USA): 5′-CATCGGAAATTTCCGGAAATTTCCGGAAATTTCCGGC-3′.

### Cell growth assay

All cell lines were seeded at a density of 5 × 10^3^ cells per well into 96-well culture plates. Following 24 hour incubation in RPMI 1640 medium with 10% fetal bovine serum, the cells were incubated for 48 hours with various concentrations of DHMEQ. To evaluate the changes of sensitivity to anticancer agents, the cells were incubated for 48 hours with various concentrations of anticancer agents (cisplatin, gemcitabine, paclitaxel and carboplatin) in each cell line. In combination analysis, cells were incubated for 48 hours with 3 μg/ml of DHMEQ and various concentrations of chemotherapeutic agents in a similar way. Cells treated with the same concentration of DMSO were served as controls. At the end of the incubation period, cell viability was determined using a Premix WST-1 Cell Proliferation Assay System (Takara Bio Inc, Shiga, Japan) and microplate spectrophotometer (Bio-Rad Laboratories, Inc, Tokyo, Japan). The absorbance value of each well was determined at 450 nm with a 655 nm reference beam in a microplate reader (Bio-Rad, Tokyo, Japan).

Resistance factor (RF) analysis also provided a qualitative measure of the extent of acquired resistance against anticancer agents, and was calculated as the half maximal (50%) inhibitory concentration (IC_50_) of resistant line/IC_50_ of parent line. IC_50_ values were determined in three independent experiments. Combination index (CI) analysis provided a qualitative measure of the extent of drug interaction. A CI of less than 1, equal to 1 and more than 1 indicates synergy, additive and antagonism, respectively [13,14].

### Apoptosis assay

Flow cytometric analysis was performed using transferase-mediated nick-end labelling (TUNEL) assay to detect apoptosis. TUNEL assay was performed using ApopTag kits (Sigma Chemical, Atlanta, GA, USA). The cells (1×10^6^ cells) were seeded in 100 mm dishes and incubated for 24 hours in RPMI 1640 medium with 10% fetal bovine serum. Following 48 hour incubation in medium containing 10 μg/ml of DHMEQ, apoptosis was detected by flow cytometry, and subsequent analysis was carried out according to the manufacturer’s protocol.

### Murine xenograft bladder cancer model

All animal procedures were carried out in accordance with ARRIVE guidelines. The protocol was approved by the Committee on the Ethics of Animal Experiments of the Keio University {Permit Number: 10228-(1)}. Six-week-old athymic nude mice (BALB/c) with an average body weight of 20 g were obtained from Sankyo Lab Service Co. (Tokyo, Japan). T24PR cells (2 × 10^6^ cells), suspended in 100 μl of matrigel (Becton Dickinson Labware, Lincoln Park, NJ, USA), were implanted subcutaneously into the flank of each mouse. In the first set of *in vivo* experiments, the mice were randomly assigned to 2 groups, each consisting of 10 animals. On day 7 after cancer cell implantation, the mice were injected intraperitoneally with 2 mg/kg DHMEQ daily. The control group was administered vehicle DMSO solution.

In the second set of *in vivo* experiments, the mice were assigned to 3 groups (control, paclitaxel alone, or combined paclitaxel and DHMEQ), each consisting of 10 animals. Paclitaxel (10 mg/kg) was administered intraperitoneally on day 14 and day 21 after cancer cell implantation, while DHMEQ was injected intraperitoneally at 2 mg/kg from day 7 after cancer cell implantation. The mice were carefully monitored and tumor size was measured every three days. Tumor volume (V) was calculated according to the formula V = length × width × height × 0.52. Four weeks after implantation, the mice were sacrificed and the tumors were evaluated histologically.

### Immunostaining for Ki-67 and apoptosis

Formalin-fixed, paraffin-embedded tissue sections (4 μm) were stained with hematoxylin and eosin (H&E) for tumor pathology. These sections were deparaffinized, rehydrated, and washed in phosphate-buffered saline. Endogenous peroxidase was quenched. A blocking step was included using 1% bovine serum albumin together with avidin and biotin blocking solutions. To determine the proliferative activity, Ki-67 immunostaining was performed using an anti-Ki-67 monoclonal antibody (MIB-1; Dako, Carpinteria, CA, USA). Apoptosis was measured by TUNEL assay using a commercially available apoptosis in situ detection kit (Wako Pure Chemical, Osaka, Japan). Visualization of the immunoreaction was performed with 0.06% 3,3′-diaminobenzidine (DAB) (Sigma Chemical, Atlanta, GA, USA). A dark accumulation of DAB in the nuclei was judged to indicate a positive reaction for TUNEL and Ki-67. The apoptotic index was calculated as the average number of TUNEL-positive cells in 10 areas at high power field (×400). The proliferation index was calculated as the average number of cancer cells with nuclei stained for Ki-67 in 10 areas at high power field (×400).

### Statistical analysis

All data are presented as the mean ± SE. Comparisons of two different groups were performed using the Mann–Whitney U-test. P-values <0.05 were accepted as being statistically significant.

Statistical analyses were performed with R Statistical Language version 2.9 and SPSS version 18.0 statistical software package.

## Results

### Efficacy of NF-κB inhibition for cell survival by DHMEQ in acquired platinum-resistant bladder cancer cells

**Figure 1:**
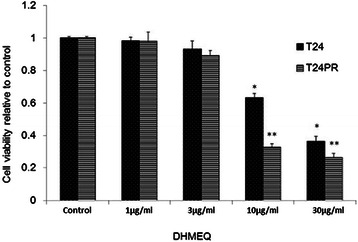
Cytotoxic effects of DHMEQ in T24 and T24PR cells. Cells were incubated for 48 hours with various concentrations of DHMEQ. Cell viability was measured by WST-1 assay. Each value represents the mean of at least 3 individual experiments; bars ± SE. *, **p < 0.05 as compared to T24 control and T24PR control, respectively.

**Figure 2:**
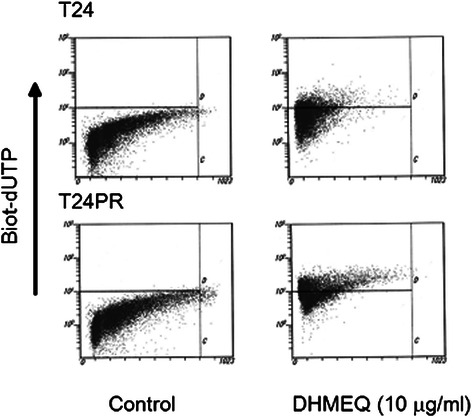
Induction of apoptosis by DHMEQ in T24 and T24 PR cells. Cells were exposed to 10 μg/ml of DHMEQ for 48 hours. TUNEL assay was performed and apoptosis was detected by flow cytometry. The upper left quadrant of each panel shows the populations of apoptotic cells.

To determine the efficacy of NF-κB inhibition by DHMEQ for cell viability, we first conducted the viability assay of T24 and T24PR cells *in vitro*. After 48 hours of incubation, DHMEQ inhibited cell growth in a dose-dependent manner in both T24 and T24PR cells (Figure 1). However, the IC_50_ of DHMEQ of T24PR cells (6.9 μg/ml) was significantly lower than that of the parent cells (17.3 μg/ml). Using the TUNEL assay, we also investigated apoptosis induced by DHMEQ (Figure 2). The apoptotic index induced by DHMEQ (10 μg/ml) at 48 hours was 23.6 ± 4.8% in T24 cells and 46.1 ± 8.5% (P < 0.05) in T24PR cells.

### Enhancement of NF-κB activity in platinum-resistant bladder cancer, and DHMEQ inhibits its activation

**Figure 3:**
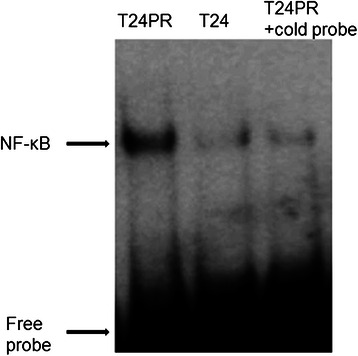
Basal NF-κB DNA binding activity in T24 and T24PR cells. Proteins were separately extracted from the cytoplasm and nucleus of T24 and T24PR cells. 2 μl nuclear extract (at a concentration of 2 μg/μl) mixed with biotin-labeled NF-κB probe for EMSA assay was used.

**Figure 4:**
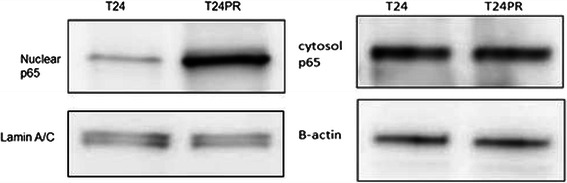
Nuclear and cytoplasm p65 protein expression in T24 and T24PR cells. The extracted nucleus and cytoplasmic protein (20 μg) were immunoblotted with p65 antibody. Lamin A/C was used as a loading control for nuclear extraction, and β-actin was used as a loading control for cytoplasm extraction.

**Figure 5:**
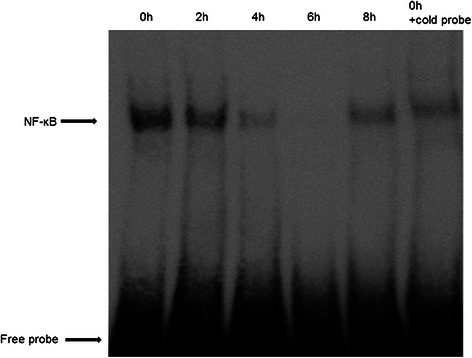
Time-dependent inhibition of NF-κB activity by DHMEQ. T24PR cells were incubated with or without 10 μg/ml of DHMEQ for various times and then the nuclear extract was assayed by EMSA.

To evaluate the changes in NF-κB expression after development of acquired platinum-resistance, we investigated the status of DNA-binding activity of p65 in T24 and T24PR cells using EMSA (Figure 3). T24PR cells exhibited strong nuclear activation of p65, whereas T24 cells exhibited weak activation. We also examined p65 protein expression in T24 and T24PR cells using Western blotting (Figure 4) and found that the nuclear p65 protein expression was apparently strong in T24PR cells as compared to T24 cells and cytoplasmic p65 levels were not different between these two cell lines. Moreover, as shown in Figure 5, the DNA-binding activity of p65 was significantly suppressed in a time-dependent manner after 2–6 hours of exposure to 10 μg/ml of DHMEQ in T24PR cells.

**Figure 6:**
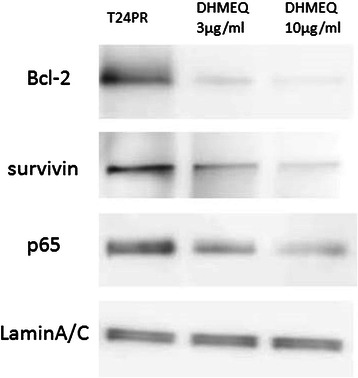
Western blotting analysis of Bcl-2, survivin and p65 in DHMEQ-treated cells. The extracted nucleus protein (20 μg) was immunoblotted with p65, Bcl-2 and survivin antibodies. Lamin A/C was used as a loading control for nuclear extraction.

We also examined nuclear protein expression Bcl-2, survivin and p65 in T24PR cells using Western blotting (Figure 6). We observed dose-dependent suppression of nuclear protein expression in Bcl-2, survivin and p65 by DHMEQ.

### Combination with DHMEQ and anticancer agents in platinum-resistant bladder cancer cells

We next examined the sensitivity of T24 and T24PR cells to anticancer agents including CDDP, gemcitabine, paclitaxel and carboplatin (Table 1). Under these experimental conditions, T24PR cells showed cross-resistance to all anticancer agents, while the sensitivity of paclitaxel did not change dramatically compared with the other agents. In the combination therapies with DHMEQ in T24PR cells, DHMEQ (3 μg/ml) could enhance the efficacy of anticancer agents. We find slightly synergistic interaction between DHMEQ and these agents and the CI values ranged between 0.7 and 0.8, respectively.

**Table 1:** **DHMEQ-mediated sensitizing effect to anticancer agents in T24 and T24PR cells**

anticancer agents	T24	T24PR		combination with DHMEQ (3 μg/ml) in T24PR	
	IC_50_ ± SE	IC_50_ ± SE	RF	IC_50_ ± SE	CI
cisplatin(μM)	4.5 ± 0.1	27 ± 1.5	5.9	9.8 ± 0.6	0.75
gemcitabine(nM)	231 ± 25	910 ± 33	3.9	124 ± 5.1	0.71
paclitaxel(nM)	49 ± 1.8	62 ± 2.6	1.3	20 ± 1.1	0.79
carboplatin(μM)	65 ± 2.8	208 ± 9.8	3.2	75 ± 5.5	0.74

Abbreviation: RF = resistance factor, CI = combination index.

**Figure 7:**
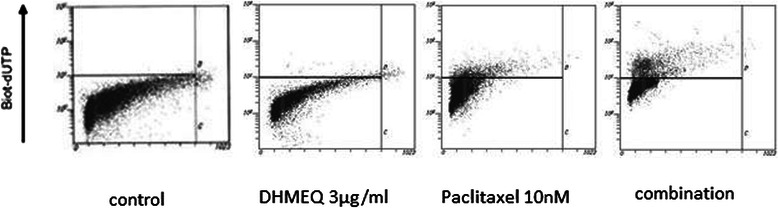
Induction of apoptosis by DHMEQ 3 μg/ml, paclitaxel 10nM, or their combination treatment. Cells were exposed to DHMEQ 3 μg/ml, paclitaxel 10nM, or their combination for 48 hours. TUNEL assay was performed and apoptosis was detected by flow cytometry. The upper left quadrant of each panel shows the populations of apoptotic cells.

**Figure 8:**
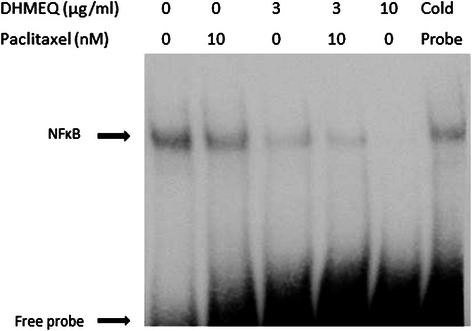
NF-κB DNA binding activity in DHMEQ 3 μg/ml, paclitaxel 10nM, or their combination treatment. Proteins were separately extracted from the cytoplasm and nucleus of each treated T24PR cells after the treatment of DHMEQ 3 μg/ml, paclitaxel 10nM, or their combination. 2 μl nuclear extract (at a concentration of 2 μg/μl) mixed with biotin-labeled NF-κB probe for EMSA assay was used.

Furthermore we evaluated the induced ability of apoptosis and DNA-binding activity of p65 when T24PR cells were treated with 3 μg/ml DHMEQ alone and the combination treatment of paclitaxel and 3 μg/ml DHMEQ *in vitro*. TUNEL assay demonstrated that the apoptotic index induced by DHMEQ (3 μg/ml), paclitaxel (10 nM), and their combination at 48 hours was 3.7 ± 0.9%, 24.8 ± 1.4% and 35.4 ± 6.1%, respectively (Figure 7). EMSA assay demonstrated that no suppression of nuclear activation of NF-κB was observed by the paclitaxel treatment alone, however, the suppression of nuclear activation by 3 μg/ml of DHMEQ was observed, and it was slightly weaker than that of 10 μg/ml of DHMEQ (Figure 8).

### Antitumor effects of DHMEQ with or without paclitaxel in a murine xenograft model of platinum-resistant bladder cancer

**Figure 9:**
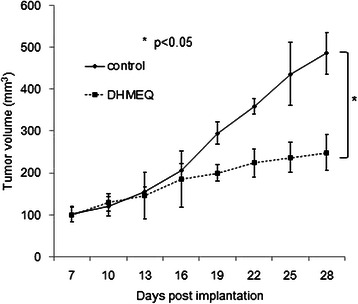
Effect of DHMEQ on tumor growth in T24PR mouse xenograft model. T24PR cells (2 × 10^6^ cells) were implanted in the flank of athymic nude mice. Seven days after implantation, daily intraperitoneal administration of 2 mg/kg of DHMEQ was started. The tumor volume of each animal was monitored and compared with that in the vehicle-treated control group. : *< 0.05, compared with control group. Each value represents the mean ± SE.

Next we examined the efficacy of DHMEQ in a murine xenograft model of the platinum-resistant subline T24PR cells. As shown in Figure 9, DHMEQ (2 mg/kg) administered intraperitoneally significantly suppressed tumor growth of the murine xenograft models of T24PR, showing the tumor volume was decreased to 51.2% compared to the control group on the 28th day. Significant differences in tumor volume were observed between the DHMEQ-treated group and control group as early as the 19th day after tumor implantation (P < 0.05).

**Figure 10:**
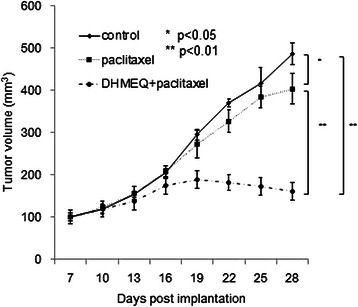
Effect of DHMEQ in combination with paclitaxel on tumor growth in T24PR mouse xenograft model. T24PR cells (2 × 10^6^ cells) were implanted in the flank of athymic nude mice. Seven days after implantation, daily intraperitoneal administration of 2 mg/kg of DHMEQ was started. Paclitaxel (10 mg/kg) was administered intraperitoneally on day 14 and day 21 after cancer cell implantation. The tumor volume of each animal was monitored and compared with that in the vehicle-treated control group or paclitaxel-treated group. : *< 0.05, **< 0.01 compared with another group. Each value represents the mean ± SE.

As shown in Table 1, the results of the cell viability assay indicated that cross-resistance to paclitaxel is relatively small in T24PR cells. We then investigated the effect of paclitaxel on a T24PR xenograft tumor model and examined whether the combination of DHMEQ with paclitaxel could enhance the therapeutic effect of the paclitaxel treatment. As shown in Figure 10, paclitaxel (10 mg/kg) was administered intraperitoneally on day 14 and day 21 after cancer cell implantation, and the mean tumor volume (403.8 ± 36.3 mm^3^) on day 28 of tumors treated with paclitaxel treatment alone was significantly smaller than those treated with vehicle control (486.3 ± 25.3 mm^3^) (p < 0.05). Furthermore, the mean tumor volume (160.8 ± 10.2 mm^3^) of tumors treated with the combination of DHMEQ and paclitaxel was significantly smaller than those treated with vehicle controls or those treated with paclitaxel treatment alone (p < 0.05, each).

### Proliferation and apoptotic index in T24PR tumors after combined therapy with DHMEQ and paclitaxel

**Figure 11:**
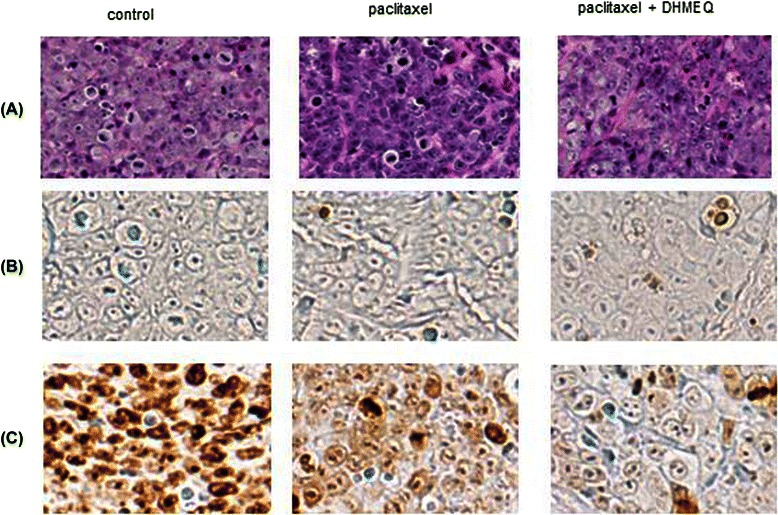
Proliferation and apoptotic index in T24PR cells treated with DHMEQ in combination with paclitaxel. Immunohistochemical study of T24PR xenograft tumors from mice treated with DHMEQ and/or paclitaxel. **(A)** Hematoxylin–eosin (HE) staining, **(B)** TUNEL staining, and **(C)** Ki-67 staining (Magnification is 1:400).

The apoptotic index of T24PR tumors was significantly increased in the paclitaxel-treated group (3.9 ± 0.9%) and combination group (10.0 ± 0.8%) compared to the control group (1.5 ± 0.3%, p < 0.05) (Figure 11, and Table 2), suggesting the apoptotic index in the combination group differed significantly from that in the paclitaxel-treated group (p < 0.05). In addition, similar results could be obtained in the analyses of the proliferation index of the tumors, showing the proliferation index in the combination group (34.3 ± 3.6%) differed significantly from that in the paclitaxel-treated group (69.1 ± 4.8%, p < 0.05) (Figure 11, and Table 2).

**Table 2:** **Suppression of tumor growth by DHMEQ in combination with paclitaxel in T24PR mouse xenograft model**

	tumor volume (mm^3^)	apoptotic index (%)	proliferation index (%)
control	486.3 ± 25.3	1.5 ± 0.3	83.7 ± 3.3
paclitaxel	403.8 ± 36.3^*^	3.9 ± 0.9^*^	69.1 ± 4.8^*^
palitaxel + DHMEQ	160.8 ± 20.3^*, **^	10.0 ± 0.8^*, **^	34.3 ± 3.6^*, **^

^*^P < 0.05 compared with control; ^**^P < 0.05 compared with paclitaxel.

## Discussion

Bladder cancer is one of the most aggressive epithelial tumors and is characterized by a high rate of early systemic dissemination. The prognosis for patients with advanced or metastatic bladder cancer remains poor [15]. The vast majority of patients treated with CDDP-based regimens develop progressive disease within 8 months of treatment, and the median survival is reported to be only 13–15 months [2,16]. Furthermore, there is still no approved treatment option for patients who develop disease recurrence or progression after CDDP-based regimens [17].

In the present study, we have demonstrated the cytotoxic effect of DHMEQ, a potent NF-κB inhibitor, after the development of acquired platinum-resistant bladder cancer cells. Strong nuclear localization of NF-κB was observed in T24PR cells whereas relatively weak nuclear expression of NF-κB was observed in T24 cells. DHMEQ reversibly inhibited the DNA-binding activity of NF-κB and consequently induced a significant dose-dependent decrease in cell viability due to apoptosis in T24PR cells. We further examined the efficacy of DHMEQ using mouse xenograft tumors, and observed a significant inhibitory effect on tumor growth especially in DHMEQ-treated tumors of T24PR cells. These data suggest that DHMEQ may be useful even in acquired platinum-resistant tumors and shed light on the impact of NF-κB inhibition as a new modality when tumors develop acquired resistance toward CDDP-treatments.

The DNA-binding activity of p65 was significantly inhibited after 2–6 hours of 10 μg/ml of DHMEQ and then gradually recovered. DHMEQ covalently binds to the cysteine residue to induce irreversible inhibition [18,19]. However, after a long incubation period, possible newly formed NF-κB appears. We think inhibition of NF-κB for several hours would be sufficient to increase the drug sensitivity. So we believe that DHMEQ has a long-lived efficacy without continuing inhibition of NF-κB translocation to the nucleus. In fact, we have examined and reported similar cytotoxic results for DHMEQ in various types of cancer cells, even though the NF-κB inhibition is short-lived [9,12,20]. Furthermore, we have shown that Bcl-2 and survivin were suppressed by DHMEQ in a dose-dependent manner. It is likely that the increase of drug sensitivity is due to the decrease of anti-apoptosis protein expression in our present study.

NF-κB activation has been found to be involved in many types of cancer including genitourinary cancer such as prostate cancer and renal cell cancer [21-23]. In bladder tumors as well, the impact of NF-κB activation on tumorigenesis has been described [24] and our previous work focused on the efficacy of NF-κB blockade by DHMEQ in a mouse xenograft model of invasive bladder cancer KU-19-19 cells [10]. Also, several researchers attempted to examine the association between the status of NF-κB expression and resistance to chemotherapy in bladder tumors. Using immunohistochemical analysis from 116 bladder cancer patients, Levidou et al. reported a close association between the aggressiveness of bladder tumors and nuclear NF-κB expression, and suggested NF-κB expression has an impact as an independent indicator for prognosis in bladder UC patients [24]. Wang et al. also reported that NF-κB activity and sensitivity to chemotherapy are inversely correlated in cancer treatments [25]. Inhibition of NF-κB not only leads to enhanced apoptosis but also to increased sensitivity to radiation or chemotherapy in several tumor cells such as fibrosarcoma and colorectal cancer cell lines as well as xenograft models or pancreatic carcinoma cells [25-27]. With regard to the association between NF-κB activation/expression and chemoresistance, Antoon et al. reported that the breast cancer chemo-resistance cell line MCF-7TN-R overexpressed NF-κB. Furthermore, inhibition of the NF-κB cascade with a sphingosine kinase-2 inhibitor decreased NF-κB activation as well as tumor growth *in vitro* and *in vivo* [26].

Paclitaxel, which is one typical taxane agent, has been used in patients with advanced urothelial carcinoma who were refractory to prior CDDP based chemotherapy. Paclitaxel alone yields a 42% response rate against urothelial carcinoma when used as a first-line treatment [27], but yields only a 10% response rate in patients who were treated previously [28]. Considering the low response rate of paclitaxel when used alone as a second-line treatment, its combinations with gemcitabine, cisplatin, carboplatin, and ifosfamide have been investigated, and the response rate was found to increase to 15–40% [28-31]. In the present study, examining further the clinical potency of DHMEQ, we investigated the efficacy of combination therapy with paclitaxel and DHMEQ in platinum-resistant tumors. Indeed, the results showed a significant difference in tumor growth between the DHMEQ-only group and combination-treated group. As shown in Figure 10, paclitaxel inhibited the growth of T24PR tumors, however, its combination with DHMEQ had a stronger antitumor effect. The reduction of tumor volume in tumors treated with the combination treatment and paclitaxel alone treatment as compared to vehicle control was 66.9% and 17.0%, respectively. Therefore, we propose that combination therapy consisting of taxane agents and DHMEQ may be an effective choice for patients with CDDP refractory bladder tumor.

## Conclusion

In summary, there was a distinct change in the expression of NF-κB between T24 and T24PR cells, suggesting strong nuclear localization of NF-κB was observed after the development of acquired platinum-resistance in bladder cancer. While NF-κB blockade leads to a significant decrease in cell viability due to apoptosis, we believe that regulation of the NF-κB pathway may be a potent therapeutic target in platinum-resistant bladder cancer.

## Abbreviations

CDDP: Cis-diamminedichloro-platinum
CI: Combination index
DHMEQ: Dehydroxymethyl derivative of epoxyquinomicin
DMSO: Dimethyl sulfoxide
EMSA: Electrophoretic mobility shift analysis
IC_50_: Half maximal (50%) inhibitory concentration
NF-κB: Nuclear factor-kappa B
RF: Resistance factor
SE: Standard error
TUNEL: Transferase-mediated nick-end labelling
